# Experience with Management of Radial Polydactyly: The Multicenter Analysis of 28 Surgical Procedures with Follow-Up

**DOI:** 10.3390/healthcare11233045

**Published:** 2023-11-26

**Authors:** David Breidung, Ioannis-Fivos Megas, Yvonne Dittrich, Bert Reichert, Ulrich Nöth, Götz Habild

**Affiliations:** 1 Department of Plastic, Reconstructive and Hand Surgery, Center for Severe Burn Injuries, Paracelsus Medical University, Klinikum Nürnberg, 90471 Nuremberg, Germany; bert.reichert@klinikum-nuernberg.de; 2 Department of Health Management, Friedrich Alexander University Erlangen-Nuernberg (FAU), 91054 Erlangen, Germany; 3 Center of Plastic Surgery, Hand Surgery and Microsurgery, Department of Orthopaedic and Trauma Surgery, Evangelisches Waldkrankenhaus Spandau, 13589 Berlin, Germany; ioannis.megas@jsd.de (I.-F.M.); yvonne.dittrich@jsd.de (Y.D.);; 4 Department of Orthopaedic and Trauma Surgery, Evangelisches Waldkrankenhaus Spandau, 13589 Berlin, Germany; ulrich.noeth@jsd.de

**Keywords:** thumb duplication, radial polydactyly, congenital abnormalities, reconstructive surgical procedures

## Abstract

Radial polydactyly or thumb duplication is a relatively common congenital malformation of the hand, whereby the surgical techniques can be broadly divided into simple excisions, reconstructions and a Bilhaut-Cloquet procedure. The aim of this study was to identify the appropriate surgical procedures and to present the clinical outcomes that can be achieved. We performed a multicenter analysis of cases of radial polydactyly surgically treated with reconstruction or a Bilhaut-Cloquet procedure between 2015 and 2022. The clinical outcome was assessed using a modification of the Tada score. A total of 28 cases of 27 patients with radial polydactyly were included in the study. The most common Wassel type was type IV (13 cases), and the most common surgical procedure was reconstruction (24 cases). Our study validates an algorithm from the literature as a helpful tool for decision making in selecting a surgical technique for radial polydactyly, although individual surgical experience should also be considered.

## 1. Introduction

Radial polydactyly or thumb duplication is a common congenital malformation of the hand. The incidence is estimated to be 0.08 per 1000 live births [1,2]. In addition to a functional deficit, which can be more or less pronounced, thumb duplications are often a cosmetic problem for the affected individuals. The sensory and motor units are often distributed between both thumbs, which often enables them to function together [3,4].

In 1969, Wassel presented a classification system for subdividing the forms of thumb polydactyly [5]. The classification is based on the level of skeletal duplication. A prediction of postoperative complications or the expectable clinical outcome; however, this is not possible with the Wassel classification [6]. In addition to the bony abnormalities in radial polydactyly, there are often deficiencies in the collateral ligaments [7]. Another classification for radial polydactyly was presented by Zuidam et al., the Rotterdam classification, which contains elements of two classification systems, the Wassel and the Buck-Gramcko classifications [5,8,9]. However, the most used classification for radial polydactyly is the Wassel classification, whereby there are discrepancies regarding the naming of the classification [10,11].

In addition to radiographic images, clinical examination factors, such as the soft tissue structures, possible hypermobility or instability of the MCP or IP joints, and the condition of the nail folds also play a role in preoperative planning [12]. Regarding surgical therapy, there are various surgical techniques available that should ultimately provide adequate joint stability, strength and mobility, as well as an acceptable aesthetic result [13]. The major surgical options include the simple excision of the hypoplastic thumb, the excision of the hypoplastic thumb with the subsequent reconstruction of the dominant thumb with parts of the excised thumb and reconstruction, according to Bilhaut-Cloquet [13]. It should be noted that the term “reconstruction”, which is often used in the literature to describe this procedure, here, refers to the process of creating a functional and aesthetically pleasing thumb by adjusting the existing tissues and not the literal meaning of “reconstruction”, which involves restoring a thumb. The reconstructive methods for radial polydactyly are diverse and range from the reconstruction of collateral ligaments to neurovascular island flap techniques [14]. In addition, various modifications of the Bilhaut-Cloquet procedure have been introduced in the literature [15,16,17]. In addition, there are also combined techniques, such as on-top plasty combined with a modified Bilhaut-Cloquet procedure, which could be relevant in complicated cases [18]. The Bilhaut-Cloquet procedure combines equal parts of the two thumbs [19]. The surgical procedure may be appropriate for more proximal thumb duplications when neither thumb provides an adequate base for reconstruction [12]. As for the timing of surgical intervention, an age of approximately 12 months is considered appropriate [20]. A treatment algorithm for radial polydactyly was presented by Dijkman et al. [21]. Here, a surgical technique was selected depending on the degree of hypoplasia of the thumbs. In unequal thumbs, where one thumb is slightly better developed than the other, resection and reconstruction should be performed, and in floating-type radial polydactyly, a simple excision should be performed. In the case of approximately and equally developed thumbs, a Bilhaut-Cloquet procedure should be performed according to the treatment algorithm. On-top plasty and pollicization should only be used in exceptional cases.

Surgical procedures run the risk of compromising the thumbs’ functionality in favor of the improved cosmetic appearance of the hand [3]. Therefore, careful surgical planning and the evaluation of the clinical outcome are key elements in the management of radial polydactyly. The postoperative outcome can be assessed using the Tada scoring system [22]. Since its introduction in 1983, this scoring system has been used in several studies to evaluate the clinical outcomes and has been modified in some cases [15,23,24]. The evaluation form of the Japanese Society for Surgery of the Hand (JSSH) is another tool used for assessing the clinical outcome [25]. Here, in addition to possible malalignment or instability, the range of movement and cosmetic appearance, possible pain and the patient’s satisfaction are also taken into account. Another scoring tool for the evaluation of clinical outcome is the Rotterdam assessment system, which was introduced by Dijkman et al. and is a modification of the JSSH evaluation form [26].

The aim of this study was to identify the appropriate surgical procedures and to present the clinical outcomes that can be achieved. For this purpose, the retrospective analysis of cases of thumb duplication was performed, and the criteria for the decision making of the surgical technique were employed.

## 2. Materials and Methods

We performed retrospective multicenter analysis. Data on surgically treated radial polydactyly were extracted from the databases of Evangelisches Waldkrankenhaus Spandau Berlin and Klinikum Nürnberg. The study period included surgeries performed between January 2015 and November 2022. Patients with thumb duplication who did not undergo surgery at Evangelisches Waldkrankenhaus Spandau Berlin or Klinikum Nürnberg were excluded from the study. Patients for whom a postoperative follow-up was not possible were also excluded from the study. The decision on an appropriate surgical procedure was made on the basis of various clinical and radiological criteria. In decision making for the Bilhaut-Cloquet procedure, one of the most important criteria was the presence of the balanced expression of thumbs. Simple excision was reserved for distal thumb duplications in which the remaining thumb had joint stability and free mobility. The patients’ files were retrospectively evaluated. Simple excisions (one case of Wassel type I) were excluded from the study to allow comparison between the two major surgical methods, reconstructions and Bilhaut-Cloquet procedure.

The relevant data for the study included age, sex, comorbidities, surgical history regarding radial polydactyly, affected hand, Wassel type, dominant side, type of surgery performed, postoperative complications, and data from the follow-up visits. During the follow-up visits, the range of motion, stability, possible malalignment, functional disturbances, problems with the pinch grip, and the scar were evaluated. Follow-up examinations were performed by different physicians. Patients with a postoperative follow-up period of less than one year were excluded from the clinical outcome evaluation. The data collected were assessed using a modification and extension of the Tada score, which has been presented by Tada et al. (see Table 1) [22]. 

Data collection and analysis were performed using Microsoft Excel. The statistical significance of differences in clinical outcomes between the different surgical procedures was not determined due to the restricted number of cases. Categorical variables were reported, with absolute numbers and percentages shown in parentheses when appropriate. Quantitative variables are reported as means with standard deviation. 

## 3. Results

A total of 28 cases of radial polydactyly surgery among 27 patients were included in the study. Of these patients, twenty-two were treated at Evangelisches Waldkrankenhaus Spandau Berlin, and five were treated at Klinikum Nürnberg. The corresponding cases are listed in Table 2, and an overview of the cases is given in Table 3. Reconstruction was the surgical procedure used in twenty-four cases (86%), and a Bilhaut-Cloquet procedure was used in four cases (14%). In the four cases in which a Bilhaut-Cloquet procedure was performed, radial polydactyly was preoperatively described as balanced. The duration of the follow-up ranged from one month to seven years. In 11 patients corresponding to 12 cases, the clinical outcome was not evaluated due to the shortness of the postoperative follow-up period. The mean mTADA score was 6.1 (SD 0.9), giving a total of fourteen cases (88%) a good score, two cases (13%) a fair score, and no patients (0%) a poor score. 

All forms of radial polydactyly, with the exception of type I, according to the Wassel classification, are represented by these cases. In the four cases of Wassel type II, three reconstructions and one Bilhaut-Cloquet procedure were performed. In all four cases of Wassel type III, reconstruction was performed. Out of 13 cases of type IV thumb duplication, 11 were treated with reconstruction, and 2 were treated with the Bilhaut-Cloquet procedure. All the cases of type V (three cases) and type VI (two cases) were treated by reconstruction. One Bilhaut-Cloquet procedure and one reconstruction were performed in the two cases of Wassel type VII. A list of the data can be found in Table 4.

The clinical outcome parameters were compared according to the surgical procedure used and the respective Wassel type (see Table 5). Sixteen patients that were assessed for the clinical outcome had an average follow-up of 2.3 ± 1.7 years. Postoperative complications did not occur in any of the cases. A total of two cases (13%) had limitations in the range of motion at the follow-up. Both the cases were Wassel type IV thumb duplications, and reconstruction was performed in both cases. Two cases (13%), one with type III as well as one with type V, had some form of postoperative instability after reconstruction. A total of two cases (13%) had a moderate malalignment, one of the cases was Wassel type II, which was reconstructed, and one case was Wassel type IV, for which the patient underwent the Bilhaut-Cloquet procedure. In one case (6%), a malalignment of 25° was observed; in this case, the Wassel type II thumb duplication was reconstructed. In one case (6%), in which a type IV thumb duplication was reconstructed, mild difficulties with coordination and pinch grip were noted during the follow-up examinations. A total of two cases (13%) of Wassel type IV, which had been reconstructed, had minor issues regarding the scar. The cases that underwent reconstruction had an average mTADA score of 6.1, while the cases that underwent the Bilhaut-Cloquet procedure had an average score of 6.3. Regarding the Wassel type, the cases with type IV thumb duplication had the lowest average mTADA score of 5.9.

## 4. Discussion

In this multicenter study, we retrospectively analyzed a total of 28 cases of radial polydactyly that were treated surgically. Overall, 88% of the patients for whom the clinical outcome could be evaluated achieved a good result based on the mTADA score, irrespective of the surgery used. There were no clear differences in the mean mTADA scores between the reconstructions and the Bilhaut-Cloquet procedure (6.1 versus 6.3).

### 4.1. Affected Patients and Types of Radial Polydactyly

The average age at surgical intervention in the cases we treated was 2.0 years, which is slightly above the recommended age of 12 months [20]. However, most of the patients were close to this age. Nevertheless, in this study, we also present a case of type IV thumb duplication that was successfully surgically reconstructed at the age of five years, and the clinical outcome was considered good, with an mTADA score of seven points. Also, a 25-year-old patient with type IV thumb duplication was treated without complications, although an evaluation using the mTADA score was not retrospectively possible. In this study, Wassel type IV thumb duplications were the most common, with thirteen cases, followed by four cases each of types II and III thumb duplications. The type IV Wassel classification is also the most common type of thumb duplication in the literature [20]. Type II is considered to be the second most common [20]. For comparison, Maillet et al. reported 14 type IV and 12 type II thumb duplications in a case series of 33 cases [27]. This demonstrates a comparable distribution to our cases, with type II being more common than it is in our study.

### 4.2. Surgical Procedures

In our study, 28 cases of radial polydactyly were treated with 24 reconstructions and four Bilhaut-Cloquet procedures. A systematic review by Miller et al. covered a total of 10 studies and 469 cases of thumb duplication [13]. With 342 cases, reconstruction was the most common surgical procedure among the included cases, as in our study. Simple excisions accounted for 79 cases, and Bilhaut procedures accounted for 48 cases. Accordingly, simple excisions were relatively more common in the systematic review than they were in our patient cohort, in which only one patient was surgically treated with this procedure, who was ultimately excluded from the study. Several studies have also reported no single case of simple excision [24,28,29]. In the study by Townsend et al., the group of patients treated with a simple excision were found to have the highest rate of re-operation, so the indication for this procedure should be considered thoroughly; it is intended for floating thumbs [3]. The classical indication for the Bilhaut-Cloquet procedure can be described as two thumbs that are balanced in their appearance, and neither thumb alone provides a solid basis for reconstruction [28]. This was also reflected in the cases we operated on using this surgical technique; in all cases, both thumbs were described as balanced in their expression. Overall, we agree with the algorithm of Dijkman et al. in the selection of the surgical technique in the management of radial polydactyly and were able to validate the algorithm in some respects based on our results [21].

### 4.3. Clinical Outcome

For the evaluation of the clinical outcomes, in addition to postoperative complications, other parameters were taken into account to form a new assessment tool, the mTADA score. This score includes components of the TADA score, which were partially modified, as well as additional assessment criteria such as the pinch grip [22]. Fourteen cases (88%) had a good outcome according to the mTADA score, while two patients (13%) had a fair clinical outcome. Due to the relatively short postoperative follow-up, we excluded 11 patients, and thereby, 12 cases from the assessment of the clinical outcome. With respect to the respective surgical techniques, the results of the mTADA score were comparable between reconstruction and the Bilhaut-Cloquet procedure (6.1 vs. 6.3, respectively). Ganley and Lubahn stated in 1995 that although the Bilhaut-Cloquet procedure can improve the cosmetic and functional outcomes, it does not achieve regular joint motion [30]. However, we cannot support this with our cases; in all the cases treated with this surgical technique, no limitation in the range of motion as well as no functional deficits were postoperatively observed. However, as our sample included only three cases of the Bilhaut-Cloquet procedure with the assessment of the clinical outcome, further studies are needed in this regard in the future. Similarly, Du et al. reported good functional results using a modified version of the BCP in a study with 24 patients [31]. The studies by Yen et al. and Ogino et al. demonstrate similar clinical outcomes for the Bilhaut-Cloquet procedure and the other surgical procedures, with Ogino et al. using a modified version of the Bilhaut-Cloquet procedure [6,13,24]. A systematic review by Miller et al. concluded that the Bilhaut-Cloquet procedure is comparable to reconstruction in terms of the clinical outcome and that both techniques have their individual benefits [13]. Regarding the Wassel types, the patients who underwent surgery for type IV thumb duplication had the lowest average score of 5.9 for the clinical outcome. In their 1996 study, Ogino et al. demonstrated worse clinical outcomes with the treatment of Wassel types III, V, and VI [24]. From our results, it can be concluded that there is no uniform consensus on which Wassel types have relatively good outcomes and which types tend to have worse expected clinical outcomes. Hovius and Kruit, in their comprehensive review, have highlighted that the most unfavorable outcomes often manifest during the growth period and may only become apparent in more extended follow-up assessments [32]. Therefore, it is important to acknowledge that, during our follow-up assessments, certain limitations in clinical outcomes may not have fully presented themselves. However, to increase the reliability of the study, we decided to limit the patients whose data were included in the assessment of the postoperative clinical outcome based on the duration of the postoperative follow-up period.

### 4.4. Strengths and Limitations

This multicenter study on radial polydactyly treatment offers valuable insights; yet, it has both strengths and limitations. The multicenter approach involves two medical centers, increasing the diversity of the patient sample and enhancing the study’s generalizability. The study provides a comprehensive overview of radial polydactyly treatments, covering the two major surgical techniques, reconstruction as well as the Bilhaut-Cloquet procedure, and Wassel types, contributing to a well-rounded understanding of the condition. Objective clinical assessments, including physical examinations during follow-up appointments, ensure a robust evaluation with objective measurements and functional evaluations. The exclusion of cases with a short postoperative follow-up allows a more reliable evaluation of the results after these operations. However, there is no clear standard for evaluating the clinical outcomes after radial polydactyly surgery, complicating the pooling of results from multiple studies [13]. This study validates the practical effectiveness of the treatment algorithm, enhancing its relevance in clinical decision making for radial polydactyly cases.

However, this study has certain limitations. The patient group’s heterogeneity in terms of age and Wassel types introduces variability and makes it challenging to draw definitive conclusions. The involvement of multiple surgeons and evaluators in the study may have contributed to challenges related to inter-rater reliability and introduced the potential for bias in the assessments. The retrospective nature of the study design resulted in certain limitations, including cases where a clinical outcome assessment was not retrospectively possible. In particular, the Bilhaut-Cloquet procedure has a limited sample size, potentially affecting the ability to detect significant differences in the outcomes. While the study provides insights up to seven years post surgery, longer-term data would be valuable to assess the durability of treatment outcomes. 

In summary, this multicenter study offers valuable insights into radial polydactyly treatment. This study therefore integrates with the existing literature and provides results with follow-up data on the surgical treatment of this condition. Future research in this field could benefit from addressing the study’s limitations, such as sample heterogeneity and the need for more extended follow-up data, to further enhance the robustness of findings and improve the clinical outcomes for the affected patients.

## 5. Conclusions

Good clinical outcomes can be achieved with the surgical treatment of radial polydactyly. Reconstruction is usually the most commonly used procedure. We were able to validate the algorithm of Dijkman et al. based on our results and support its use to select the surgical technique based on the degree of hypoplasia of the thumbs. In addition, the surgeon’s individual experience should be considered during surgical planning, and good results can be achieved both functionally and aesthetically with different techniques.

## Figures and Tables

**Table 1:** The mTADA score as an assessment tool for follow-up findings was developed as a modification and extension of the Tada score. A maximum score of 7 points can be achieved, with a score of 7 or 6 representing a good result, a score of 5 or 4 representing a fair result, and a score below 4 representing a poor result.

Parameters	Description	Score
Overall range of motion in IP + MCP joint	More than 70°	1
	Less than 70°	0
Instability	Absent	1
	Instability	0
Malalignment	Absent or less than 10°	2
	10°–20°	1
	More than 20°	0
Functional deficit	Absent	1
	Functional deficit	0
Pinch Grip	No abnormalities	1
	Difficulties related to pinch grip	0
Scar	Inconspicuous scar	1
	Scar problems	0

IP: interphalangeal; MCP: metacarpophalangeal.

**Table 2:** Demographics, comorbidities, data concerning the radial polydactyly, type of surgery, duration of follow up and mTada-score of radial polydactyly cases.

Age	Sex	Comorbidities	Affected Hand	Wassel Type	Dominant Side	Type of Surgery	Duration of Follow-Up	mTada Score
11	m		Right	IV	Ulnar	REC	4 years	6
12	w		Left	II	Balanced	BCP	3 years	6
16	w	Surgically treated coarctation of aorta, ventricular and atrial septal defect	Left	IV	Ulnar	REC	2 years	4
14	w		Right	VI	Radial	REC	4 months	
13	m		Right	II	Balanced	REC	3 months	
11	m		Right	V	Ulnar	REC	2 years	7
15	w		Right	IV	Balanced	BCP	1 year	6
14	m		Right	III	Balanced	REC	5 years	6
75	w		Right	IV	Ulnar	REC	1 month	
11	w		Right	V	Ulnar	REC	6 months	
17	m	Failure to thrive, iron deficiency anemia	Left	IV	Balanced	BCP	1 year	7
14	m	Intellectual disability	Left	IV	Ulnar	REC	2 years	6
23	w		Right	IV	Ulnar	REC	1 month	
14	w		Both	R: VII L: VII	R: Balanced L: Ulnar	R: BCP L: REC	2 months	
12	w		Right	IV	Ulnar	REC	2 months	
70	w		Right	IV	Balanced	REC	1.5 years	7
17	w		Right	II	Ulnar	REC	3 years	5
17	w		Left	III	Ulnar	REC	1.5 years	7
22	m		Left	III	Balanced	REC	1 year	7
13	m		Left	IV	Ulnar	REC	1 year	6
18	w		Left	II	Ulnar	REC	1 year	6
11	w		Right	IV	Radial	REC	2 months	
25	m		Right	IV	Ulnar	REC	1 year	7
12	m		Left	III	Ulnar	REC	1 month	
13	w		Right	VI	Ulnar	REC	1 month	
12	m		Right	V	Ulnar	REC	7 years	6
271	m		Right	IV	Ulnar	REC	1 month	

REC: Reconstruction, BCP: Bilhaut-Cloquet procedure.

**Table 3:** Overview of radial polydactyly cases.

Patients (n = 27) and Cases (n = 28) with Radial Polydactyly	
Age, years	2.0 ± 4.2
Gender	
Male	12 (44%)
Female	15 (56%)
Comorbidities and risk factors	
Prior medical conditions	3 (11%)
No prior medical conditions	24 (89%)
Affected hand	
Right	17 (63%)
Left	9 (33%)
Bilateral	1 (4%)
Wassel type	
I	0 (0%)
II	4 (14%)
III	4 (14%)
IV	13 (46%)
V	3 (11%)
VI	2 (7%)
VII	2 (7%)
Dominant side	
Radial	17 (61%)
Ulnar	3 (11%)
Balanced	8 (29%)
Duration of follow-up, years	1.5 ± 1.7

**Table 4:** Performed surgical procedures for thumb duplications subdivided by Wassel type.

Wassel Type	Reconstruction	Bilhaut-Cloquet Procedure
I	0 (0%)	0 (0%)
II	3 (75%)	1 (25%)
III	4 (100%)	0 (0%)
IV	11 (85%)	2 (15%)
V	3 (100%)	0 (0%)
VI	2 (100%)	0 (0%)
VII	1 (50%)	1 (50%)

**Table 5:** Clinical outcome parameters for different surgical procedures and Wassel types.

Surgical Procedures/Wassel Types	Cx	ROM	Instability	Malalignment	Functional Deficit	Pinch Grip	Scar	mTada Score
Reconstruction	0 (0%)	0.8 ± 0.5	0.8 ± 0.4	1.7 ± 0.4	0.9 ± 0.3	0.9 ± 0.3	0.8 ± 0.4	6.1 ± 1.0
Bilhaut-Cloquet procedure	0 (0%)	1.0 ± 0.0	1.0 ± 0.0	1.3 ± 0.6	1.0 ± 0.0	1.0 ± 0.0	1.0 ± 0.0	6.3 ± 0.6
Wassel type II	0 (0%)	1.0 ± 0.0	1.0 ± 0.0	1.0 ± 1.0	1.0 ± 0.0	1.0 ± 0.0	1.0 ± 0.0	6.0 ± 1.0
Wassel type III	0 (0%)	1.0 ± 0.0	0.7 ± 0.6	2.0 ± 0.0	1.0 ± 0.0	1.0 ± 0.0	1.0 ± 0.0	6.7 ± 0.6
Wassel type IV	0 (0%)	0.8 ± 0.5	1.0 ± 0.0	1.6 ± 0.7	0.9 ± 0.4	0.9 ± 0.4	0.8 ± 0.5	5.9 ± 1.0
Wassel type V	0 (0%)	1.0 ± 0.0	0.5 ± 0.7	2.0 ± 0.0	1.0 ± 0.0	1.0 ± 0.0	1.0 ± 0.0	6.5 ± 0.7

Cx: complications; ROM: overall range of motion in IP + MCP joint.

## Data Availability

Data are contained within the article.
